# Growth, production and feed conversion performance of the gurami sago (Osphronemus goramy Lacepède, 1801) strain in different aquaculture systems

**DOI:** 10.12688/f1000research.22201.3

**Published:** 2021-01-27

**Authors:** Azrita, Netti Aryani, Ainul Mardiah, Hafrijal Syandri

**Affiliations:** 1 Department of Biology Education, Faculty of Education, Bung Hatta University, Padang, 25133, Indonesia; 2 Department of Aquaculture, Faculty of Fisheries and Marine Science, Riau University, Pekanbaru, 28293, Indonesia; 3 Department of Aquaculture, Faculty of Fisheries and Marine Science, Nahdlatul Ulama University of West Sumatera, Padang, 25176, Indonesia; 4 Department of Aquaculture, Faculty of Fisheries and Marine Science, Bung Hatta University, Padang, 25133, Indonesia

**Keywords:** Giant gourami, aquaculture systems, juveniles, growth, environment factors

## Abstract

**Background:** Giant gourami ( *Osphronemus goramy, Osphronemidae*), belonging to gurami sago strain, is an important economic fish species that was newly released for domestication in 2018 in Indonesia. The present study aimed to determine the growth, production and feed conversion efficiency of gurami sago strain in different aquaculture systems.

**Methods: **A mean of 240 juveniles were stocked (initial weight mean, 54.53 g and length 13.88 cm) into concrete ponds, ﬂoating net cages and earthen freshwater ponds (12 m ^3^) with three replicates of each. The juveniles were fed a floating commercial pellet diet containing 30% crude protein and 5% crude lipids. Feed was supplied at 3% of fish biomass per day throughout the 90 days of the experiment. The research was conducted in the area surrounding Lake Maninjau of Indonesia.

**Results: **After 90 days, the mean weight of ﬁsh reared in concrete ponds was 166.86 g, floating net cages was 179.51 g and earthen freshwater ponds was 149.89 g. The mean final biomass was 37.64 kg for concrete ponds, 41.27 kg for floating net cages, and 33.72 kg for earthen freshwater ponds. The specific growth rates (%/day) for concrete ponds, floating net cages and earthen freshwater ponds were 0.67, 0.75 and 0.62, respectively. The feed conversion rates were 1.45 for concrete ponds, 1.30 for floating net cages and 1.87 for earthen freshwater ponds. The net yields (kg m ^ˉ3^) were 2.05 for concrete ponds, 2.27 for floating net cages, and 1.73 for earthen freshwater ponds. The exponents (b) of the length–weight relationship were calculated for concrete ponds (1.0146), floating net cages (1.2641), and earthen freshwater ponds (1.0056).

**Conclusion: **The study showed that the growth performance, production and feed conversion efficiency of the gurami sago strain were the best found in floating net cages and considered a new aquaculture system in the future.

## Introduction

Aquaculture activities have been responsible for the supply of fish for human consumption. To meet the demand for food from aquaculture production arises competition use natural resources, such as land and water ^1– 3^, included species, and aquaculture system ^4– 6^.

The giant gourami *Osphronemus goramy* Lacepède (1801) is one of the main freshwater commodities of economic importance. This species has been produced in small-scale farms for decades in Indonesia ^7, 8^. However, only contributed as much 6.96% of the total freshwater aquaculture production. Meanwhile, *Nile tilapia*, *Clarias* catfish, *Pangasius* catfish, and common carp has been contributed 37.93%, 33.35%, 12.38%, and 9.28% of 3,374,924 metric tons freshwater fish production ^9^. Therefore, there are still important gaps of knowledge in its aquaculture of giant gourami.

Although the contribution from giant gourami was lowest (6.96%), but the local gurami tambago and gurami galunggung strain have been cultured in semi-intensive ^7, 8^. The giant gourami belongs to the local gurami sago strain has never been cultured intensively. This species is the result of newly released domestication in 2018 ^10^, which still limited in West Sumatera Province of Indonesia ^11^. Gurami sago is an herbivorous species which can consume a variety of plants such as sente leaves ( *Alocasia macrorrhiza*), kale ( *Brassica oleracea*), cassava leaves ( *Manihot esculenta*), and others young land plants. In addition, this species can eat commercial pellets, and tolerate crowded aquaculture production systems, such as earthen freshwater ponds and artificial ponds lined with membranes ^8, 11, 12^.

The gurami sago strain has been detected as candidate species for production in middle-scale farm in Indonesia ^11^. This species grows well in nursery ponds and reach a market size of 200 to 300 g per fish and a size of 50 to 100 g per fish as ornamental fishes. This characteristic creates commercial interest as a new species in an effort to develop freshwater fish farming in the future. Concrete ponds and floating net cages were options in the development of gurami sago culture. Many studies have found that continuous water flow systems in concrete ponds, artificial ponds lined with membranes, tanks, canvas tanks, pens and many other systems could be an alternative for fish aquaculture because these systems provide a high degree of control that can allow for high production ^5, 12– 16^.

In the last decade, cage systems have received more attention from both researchers and producers. Fish farming in cages can be practiced intensively ^17, 18^. High production can be achieved at a low cost ^19, 20^. Fish farming in cages can achieve maximum growth with a high survival rate ^18, 21, 22^. However, cage fish farming has advantages and disadvantages that must be considered before choosing a production system. The main disadvantages of fish farming in the floating net cages of lakes are that they are not ideal for land use and may cause massive fish deaths ^23, 24^. Meanwhile, the advantages of floating net cage aquaculture include high water circulation, solid waste not accumulating near cages, low water quality variation, and no electrical power required for water aeration ^18, 21, 22, 25, 26^.

Fish production systems in many countries use a variety of methods, e.g., carp in earthen freshwater ponds ^27^, giant gourami in earthen freshwater ponds and artificial ponds lined with membranes ^7, 12^. *Nile tilapia* in the ponds and cages ^28^, and golden pompano in the floating cages ^29^. Because the rearing of the gurami sago strain is relatively new, there are no parameters or best methods available to predict the growth performance, survival and feed conversion efficiency in a commercial rearing system. Therefore, knowledge about the contribution of gurami sago to each aquaculture system is very important to analyze. The current study was conducted to assess the growth performance, production, economic food conversion rate and waste load of feed of gurami sago strains in different aquaculture systems namely, concrete ponds, floating net cages and earthen freshwater ponds.

## Methods

### Ethical considerations

There are no required permits from the government of the Republic of Indonesia to culture the gurami sago ( *O.goramy*) strain in this study in concrete ponds, floating net cages and earthen freshwater ponds in the area surrounding Lake Maninjau of West Sumatera Province of Indonesia. The study was founded by LPPM (Research and Community Service) University of Bung Hatta under the Indonesia Endowment Fund for Education, Ministry of Finance, Republic of Indonesia, through the competitive grants scheme called the Productive Innovative Research (Policy/Governance) 2019 with the contract number PRJ-99/LPDP/2019. This grant included ethical approval and permits to collect fish samples including permission to rear this species. The animals used in this study did not suffer during the experiment. Gurami sago was transported to concrete ponds, floating net cages and earthen freshwater ponds for rearing for 90 days, fed commercial pellets and measured for growth performance every 30 days. At the end of the experiment, the gurami sago were still in good condition.

### Study area

The study was conducted at the Research Center of Faculty of Fisheries and Marine Science, Bung Hatta University located in the area of Lake Maninjau, Koto Malintang village, Tanjung Raya sub-district, District Agam of West Sumatera Province, Indonesia. The geographical coordinates were S:00°12'26.63"-S:00°25'02.80" and E:100°07'43.74"-E:100° 16'22.48" and the altitude was 461 m above sea level. At the location, concrete ponds, earthen freshwater ponds and floating net cages were available.

### Experimental design

Each concrete pond has a size of 4×2 m, a depth of 1.5 m and a volume of 12 m ^3^. It has 50 mm of middle drainage, which is covered with a net of 0.5 cm mesh to prevent juveniles from escaping and predators from entering. The water was pumped from borehole wells at a velocity of 5 litres per minute.

Each floating net cage has a size 4 × 2 m, a depth of 1.5 m and a volume of 12 m ^3^, and these cages were built from resistant PVC plastic. Each cage was constructed using a monofilament net with 10 mm mesh. The floating net cages were set up in Lake Maninjau near the fish farm (maximum depth of 9 m and an average water current of 25 cm per sec). The surface of the floating net cages was covered with nets stretched (25 mm mesh) to avoid bird predators.

Each earthen freshwater pond has a size of 4 × 2 m, a depth of 1.5 m and a volume of 12 m ^3^. It had 50 mm of central drainage and was covered with a net of 0.5 cm mesh to prevent fish jumping and predator entry during the rearing activity. The water was pumped from wells at a velocity of 5 litres per minute.

### Sampling design

The experiment ran for 90 days beginning on 01 April and ending on 29 June 2019. Approximately 3,000 gurami sago juveniles weighing approximately 50 g were obtained from a hatchery in the Luhak sub-district in the district of Lima Puluh Kota. Fish were acclimatized with 1000 juveniles per each pond (concrete pond, floating net cages and earthen freshwater pond). Fish were acclimatized to the floating net cages (5 × 5 × 3 m) for one month prior to the experiment. In the initial growth phase, three concrete ponds, three floating net cages and three earthen freshwater ponds of 12 m ^3^ (three replicates) were stocked with 240 juveniles each, with a density of approximately 20 fish/m ^3^. The average initial weights and lengths of juveniles were 54.51±0.45 g and 13.81±0.02 cm (mean ± SD), respectively. The length was measured using a ruler with an accuracy level of 0.1 cm. The weight of each individual was measured with an electronic balance (OHAUS, Model CT 1200-S, USA).

Fish were fed twice daily (09:00 AM and 17:00 PM) with commercial floating pellet feed (JapfaComfeed Indonesia Ltd; 30% crude protein, 5% crude lipids, 6% crude ash and 13% crude fibre) ^18^. The amount of feed provided was as much as 3% per day based on fish biomass during the experiment. Every 30 days, samples were taken from ponds to monitor fish growth and to adjust the feed amount. Twenty-four fish samples were obtained from each concrete pond, floating net cage and earthen freshwater pond. 10% of the fish were sampled every month for each aquaculture system, due to giant gourami is sensitive to handling. Fish were captured at 07.00 AM with gillnets, which have a net bag with a suitable mesh size. Then, fish were anaesthetized orally with tricaine methanesulfonate (MS-222, ethyl 4-aminobenzoate methanesulfonate 98%, Sigma Aldrich Co, USA, MO; 50 mg L ^ˉ1^), based on the dosage used for *Hemibagrus wyckii* ^30^.

### Water quality

Water parameters were recorded weekly in the concrete ponds, floating net cages and earthen freshwater ponds. The water temperature ( ^⍛^C) and dissolved oxygen (DO; mg L ^ˉ1^) were measured with an oxygen metre (YSI model 85). The pH values were determined using a pH metre (Digital Mini-pH Metre, 0-14PH, IQ Scientific, Chemo-science (Thailand) Co., Ltd, Thailand). The levels of ammonia (NH _3_; mg.L ^ˉ1^), nitrite-nitrogen (NO _2_-N; mg L ^ˉ1^), nitrate-nitrogen (NO _3_-N; mg L ^ˉ1^), chemical oxygen demand (COD; mg L ^ˉ1^), biological oxygen demand (BOD _5_; mg L ^ˉ1^), alkalinity (mg L ^ˉ1^), hardness (mg L ^ˉ1^), total dissolved solids (TDS; mg L ^ˉ1^) and total suspended solids (TSS; mg L ^ˉ1^) were measured in each aquaculture system with replication according to standard procedures ^31^. The nets of the floating cages were cleaned routinely to maintain water circulation in the fish rearing areas. The walls of the floating net cages were cleaned by divers in the water.

### Measurement parameters

The gurami sago were reared for 90 days, and the survival rate was estimated by checking the aquaculture systems every day and recording the results. Dead fish were removed immediately. The survival rate percentage was calculated by subtracting the number of dead fish from the initial number of the stock. The parameters were analyzed according to Aryani *et al*. ^8^, Kibra and Haque ^27^ and Mokoro *et al*. ^32^ with the following equations:

- Absolute growth rate (AGR; g day ^ˉ1^) or ( *Wt*- *Wi*)/ *t*, where *Wt* = final weight, *Wi* = initial weight, and *t* = time (day);
- Specific growth rate (SGR, % day ^ˉ1^) = ( *ln*W _1_- *ln*W _2_/ *t* × 100)
- Gross yield (kg m ^ˉ3^) = total number of fish at harvest × average final weight/cage capacity
- Net yield (kg m ^ˉ3^) = (harvested biomass - stocked biomass/cage capacity)
- Feed conversion efficiency (FCE) = [fish weight gain (g)/total feed ingested (g)]
- Apparent feed conversion rate (AFCR) = supplied feed/increase fish weight
- Economic AFCR = cost/kg of fish weight × feed cost
- Waste load of feed = [feed intake (kg)] × [waste load/kg of feed]

For each aquaculture system, the final total length (cm) and final total weight (g) were used to determine the relationship of *W = aL ^b^,* where *W* is the total wet weight (g), *L* is the total length (cm) and *a* and *b* are variables of the length–weight relationships (LWRs) equations. These variables were estimated by the least square regression method. A t-test was used for comparison of the *b* values obtained in the linear regressions with the isometric value by equation ^33^: *t _s_* = ( *b* – 3)/ *S _b_*, where *t _s_* is the t-test value, *b* is the slope and *S _b_* is the standard error of the slope ( *b*). The comparison of the obtained values of the t-test with the respective table critical values allowed for the determination of whether the *b* values were statistically significant as well as their inclusion in the isometric range ( *b*=3) or allometric range (negative allometric; *b*<3 or positive allometric; *b*>3). The degree of correlation between the variables was computed to determine the coefficient, R ^2^. Fulton’s condition index was calculated as *K=100(W/L ^3^)* ^33^, where K = Fulton’s condition index, W = weight, and L= length.

### Data analysis

The data were analyzed using SPSS software (version 16.0 for Windows; SPSS Inc., Chicago, IL). Kolmogorov-Smirnov statistics were used to test data normality. Then, Levine’s test was used to analyse the absolute residuals from homogeneity. One-way ANOVA was used to analyze the effect of each treatment, followed by post hoc Duncan’s multiple range tests ^34^. The 95% confidence level (p<0.05) was considered as the threshold to identify significant differences. All means are given with ± standard deviation (±SD). The canonical discriminant functions were used to analyze the water quality grouping between rearing systems.

## Results

The overall survival rate of fish in different aquaculture systems was greater than 89.44%. The culture system had a significant effect (p<0.05) on the mean final body weight (g), final biomass (kg), weight gain (g), gross yield (kg m ^ˉ3^), net yield (kg m ^ˉ3^), absolute growth rate (g day ^ˉ1^), specific growth rate (% day ^ˉ1^), AFCR, and economic food conversion rate (US$/kg gain) after 90 days of culture ( Table 1). In contrast, the culture system did not significantly (p>0.05) affect the mean final total length, feed intake (kg) or Fulton’s K. The economic AFCRs were US$1.45 for concrete ponds, US$1.30 for floating net cages and US$1.87 for earthen freshwater ponds.

**Table 1.** Growth performance of gurami sago in three aquaculture systems over 90 days.

Variable	Aquaculture system mean ± SD		
	Concrete ponds	Floating net cages	Earthen freshwater ponds
Mean initial TL (cm)	13.81±0.02	13.88±0.02	13.88±0.02
Mean ﬁnal TL (cm)	19.87±1.05	22.49±2.41	19.93±1.73
Mean initial body weight (g)	54.53±0.09	54.53±0.32	54.54±0.53
Mean ﬁnal body weight (g)	166.86±7.95 ^a^	179.51±2.52 ^b^	149.89±4.79 ^c^
Initial biomass (kg)	13.00±0.11	12.97±0.10	13.00±0.10
Final biomass (kg)	37.64±1.51 ^a^	41.27±0.35 ^b^	33.72±0.78 ^c^
Weight gain (g)	114.47±4.80 ^a^	125.47±2.43 ^b^	102.88±0.92 ^c^
Gross yield (kg m ^-3^)	3.14±0.13 ^a^	3.36±0.09 ^b^	2.81±0.07 ^c^
Net yield (kg m ^-3^)	2.05±0.13 ^a^	2.27±0.08 ^b^	1.73±0.07 ^c^
Absolute growth rate (g day ^-1^)	1.27±0.05 ^a^	1.39±0.03 ^b^	1.14±0.01 ^c^
Speciﬁc growth rate (% day ^-1^)	0.67±0.05 ^a^	0.75±0.02 ^b^	0.62±0.01 ^c^
Feed intake (kg)	52.62±0.14	59.24±0.14	50.21±0.49
Apparent food conversion rate	1.45±0.03 ^a^	1.30±0.02 ^b^	1.87±0.14 ^c^
Economic food conversion rate (US$/kg gain) *	1.24±0.06 ^a^	1.00±0.02 ^b^	2.08±0.30 ^c^
Condition factor (Fulton’s K)	2.45±0.63	1.91±0.01	3.36±0.05
Survival (%)	92.92±1.50	95.42±1.25	89.44±1.88
Feed conversion efficiency	0.69±0.02 ^a^	0.77±0.01 ^b^	0.54±0.04 ^c^
Waste load/kg of feed	0.31±0.02	0.23±0.01	0.46±0.04
Waste load of feed (kg)	16.22±0.90 ^a^	13.51±0.65 ^b^	23.28±2.31 ^c^

Within a row, means followed by different letters are significantly different ( *p*<0.05). TL: total length. *USD 1.00 = IDR 14,350.

**Figure 1.**
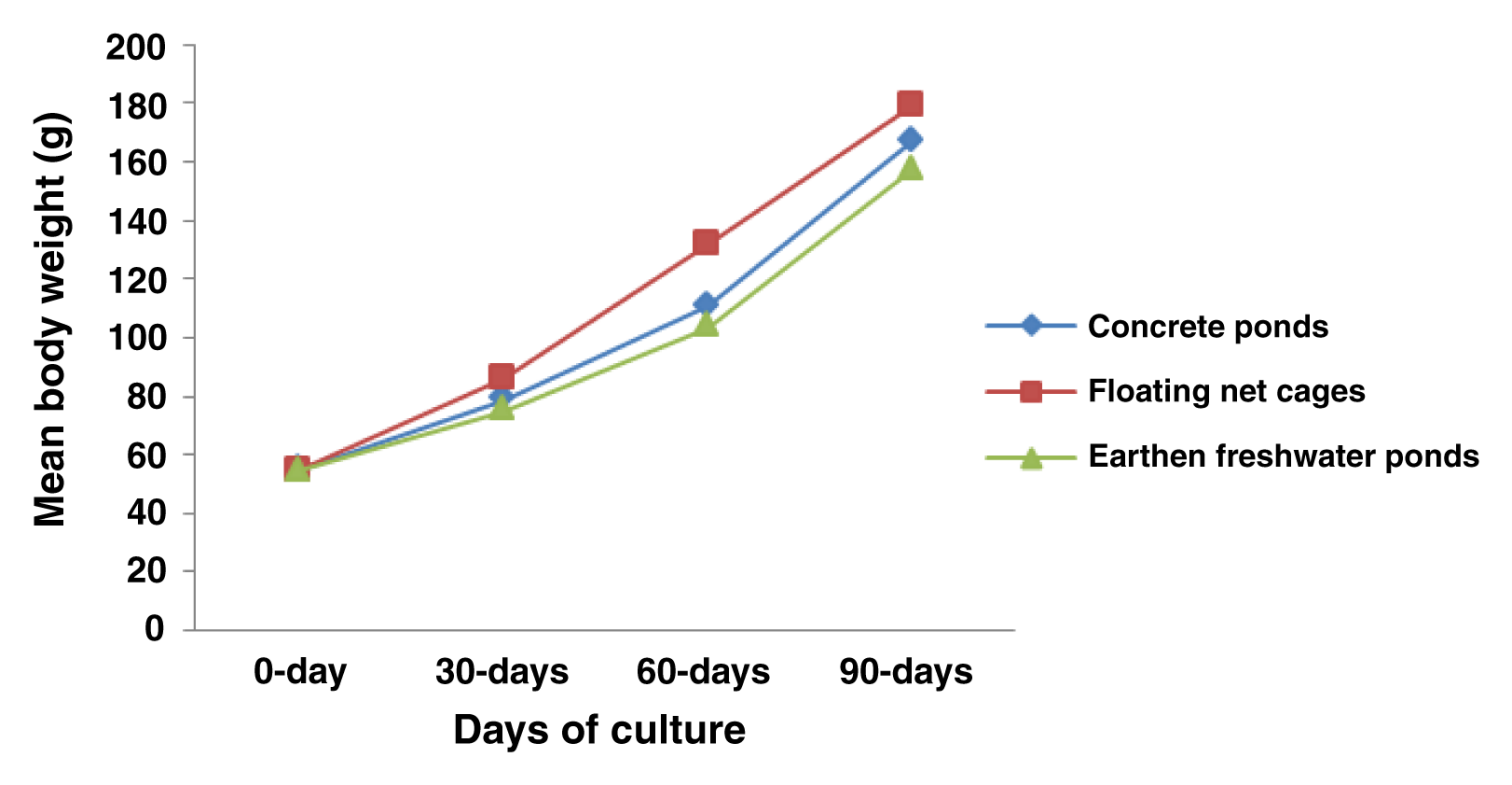
Mean weight gain ± SD (g) of gurami sago in three different aquaculture systems.

**Figure 2.**
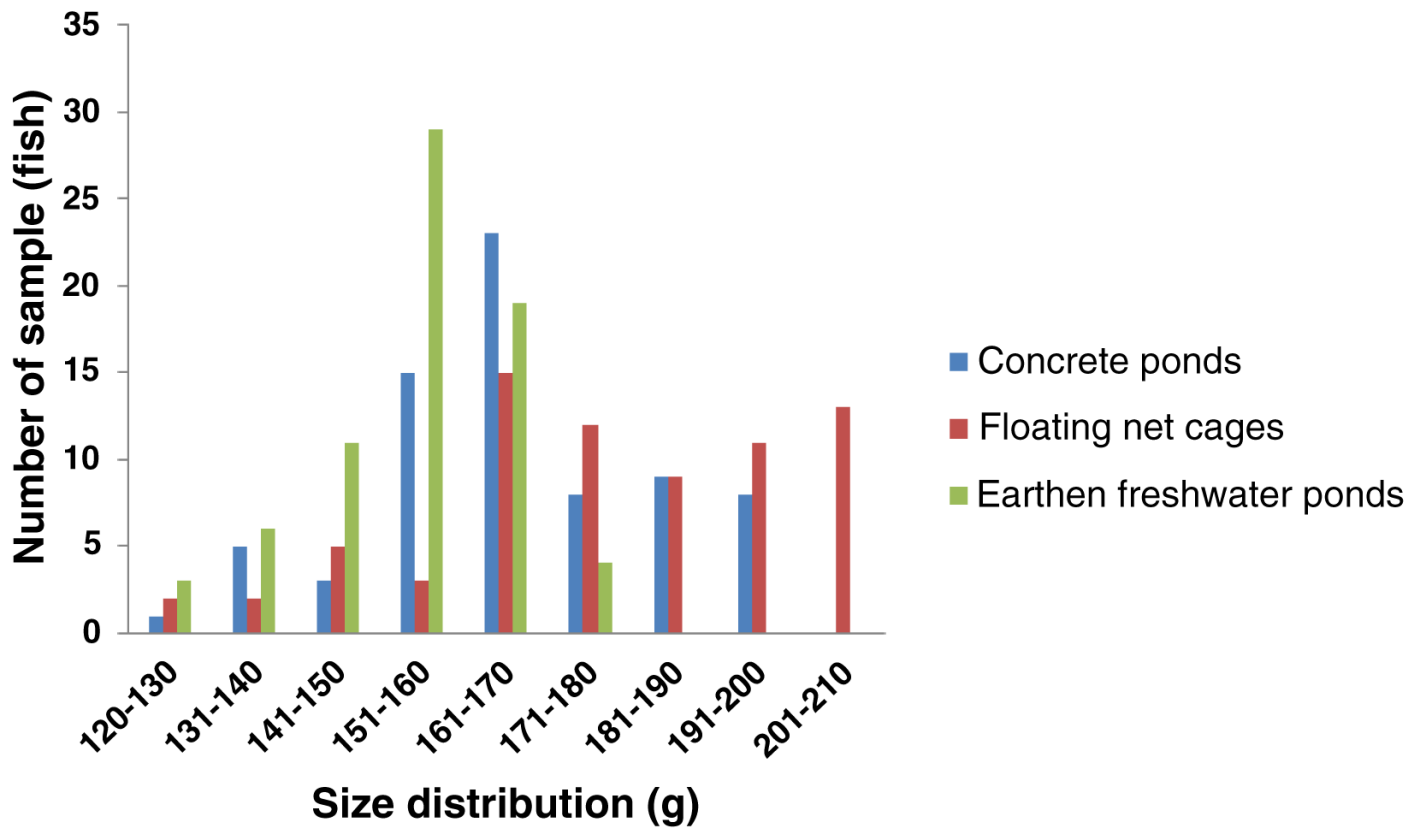
Distribution of gurami sago in the different aquaculture systems ( *N*=72).

During the 90 days of the experiment, the gurami sago reared in floating net cages grew faster than those reared in concrete ponds and earthen freshwater ponds ( Figure 1). At the end of the experiment, the fish reared in the floating net cages had a larger size distribution than that of the fish reared in the concrete ponds and earthen freshwater ponds throughout the 90 day trial ( Figure 2). The mean final body weights of the gurami sago reared in concrete ponds, floating net cages and earthen freshwater ponds were 166.86 g, 179.51 g, and 149.89 g, respectively. The net yield was 2.05 kg m ^ˉ3^ for concrete ponds, 2.27 kg mˉ ^**3**^ for floating net cages and 1.73 kg m ^ˉ3^ for earthen freshwater ponds during the 90 days of rearing. The FCE and waste load at 90 days of culture were significantly (p<0.05) affected by the different rearing systems. A summary of the FCR, FCE and waste load feed from the five aquaculture species is presented in Table 2.

**Table 2.** Data on FCR, FCE and waste load from producing 1 kg feed in five aquaculture species.

Species	Scientiﬁc name	Production system	FCR	FCE	Waste load	Reference
Giant gourami	*Osphronemus goramy*	Floating cage	1.30	0.77	0.23	This study
Tilapia	*Oreochromis niloticus*	Floating cage	1.70	0.59	0.41	Chiu *et al*., ^35^
Spotted rose snapper	*Lutjanus guttatus*	Floating cage	1.44	0.69	0.31	Hernández *et al*., ^36^
Golden pompano	*Trachinotus ovatus*	Floating cage	1.53	0.65	0.35	Qi *et al*., ^29^
Common carp	*Cyprinus carpio*	Floating cage	2.10	0.47	0.53	Mungkung *et al*., ^37^

The FCE for giant gourami culture is 0.77 (1.0 kg feed fish results in 0.77 kg of fish). This value suggests that the waste load is 0.23 kg (1.0 kg feed – 0.77 kg fish). The above calculation can be applied to other species. FCR, feed conversion rate; FCE, feed conversion efficiency.

**Figure 3.**
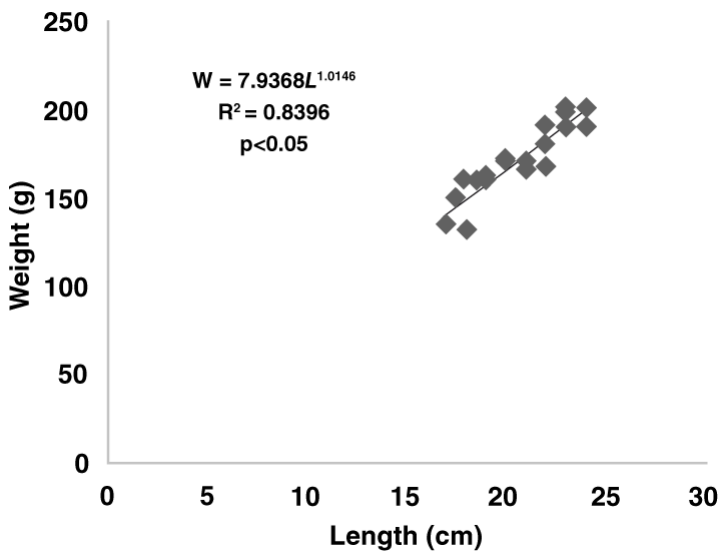
Total length-weight relationship for gurami sago cultured in concrete ponds. Each point represents one sampled fish ( *N*=24). The regression equation, coefficient of determination (R ^2^) and significance ( *p*-values) are also provided.

**Figure 4.**
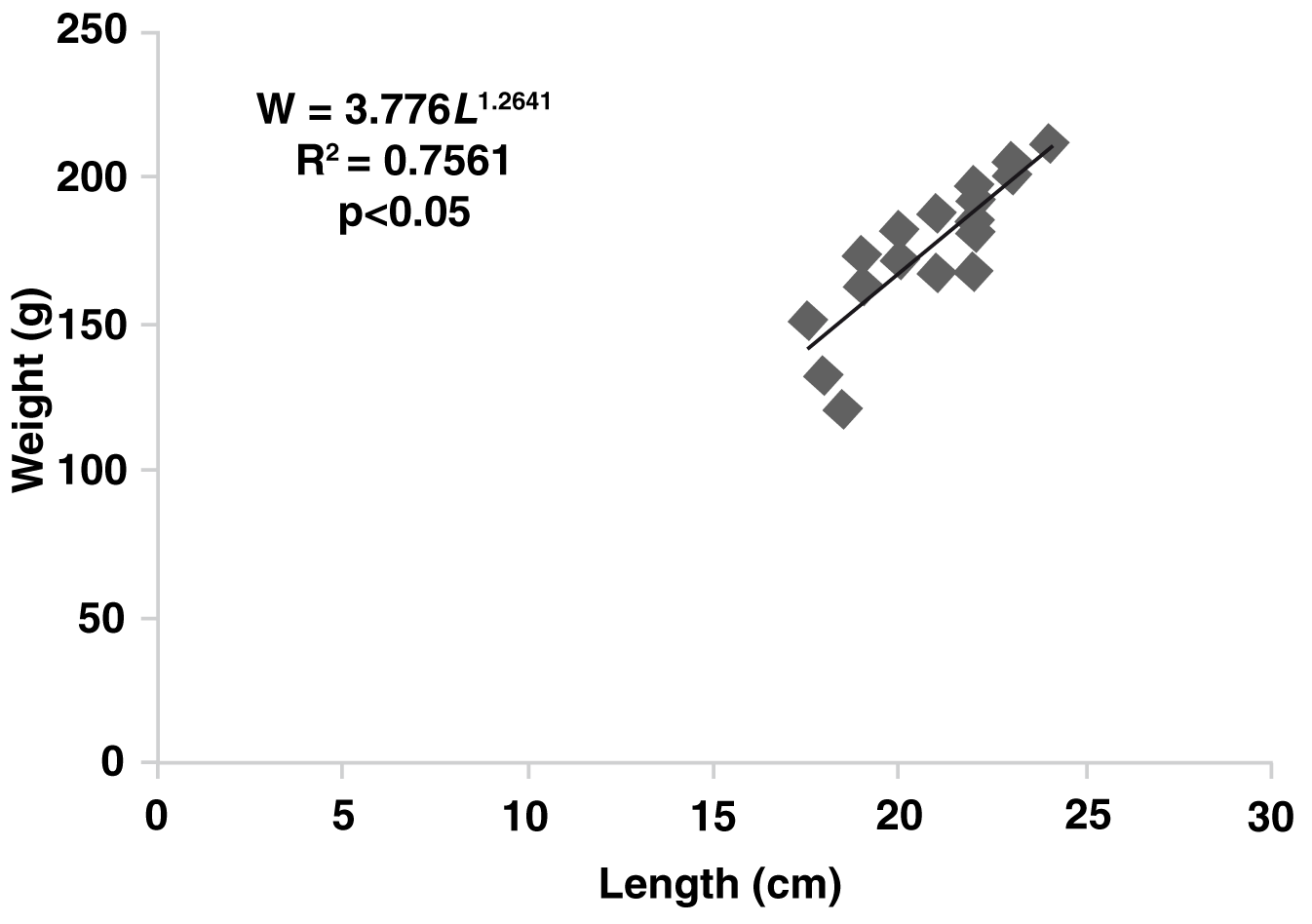
Total length–weight relationship for gurami sago cultured in floating net cages. Each point represents one sampled ﬁsh ( *N*=24). The regression equation, coefficient of determination (R ^2^) and significance ( *p*-values) are also provided.

**Figure 5.**
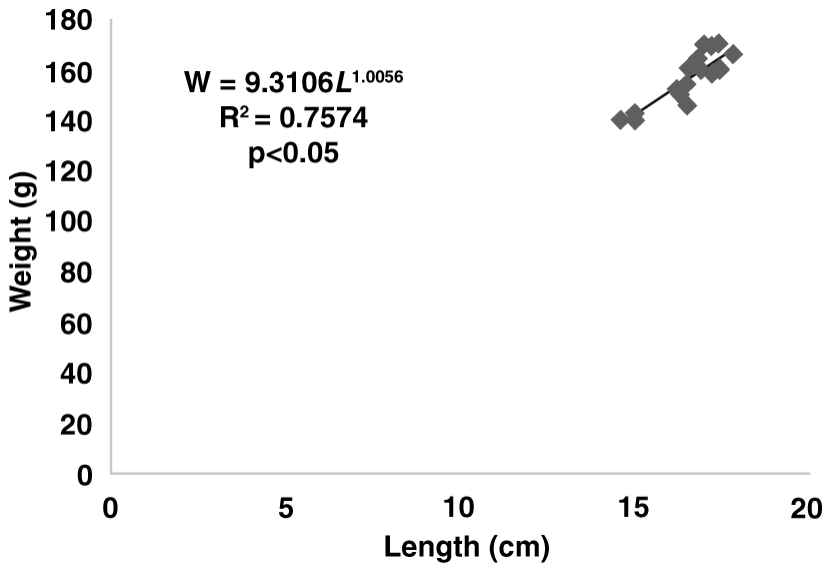
Total length-weight relationship for gurami sago cultured in earthen freshwater ponds. Each point represents one sampled ﬁsh ( *N*=24). The regression equation, coefficient of determination (R ^2^) and significance ( *p*-values) are also provided.

The growth rates of gurami sago based on body weight were described according to the following exponential equation: *W* = 60.875e ^0.0498 *t*^ (with *R* ^2^ = 0.83) for the concrete pond, *W* = 48.580e ^0.0613 *t*^ (with *R* ^2^ = 0.75) for the floating net cage and *W* = 55.7050e ^0.0623 *t*^ (with *R* ^2^ = 0.75) for the earthen freshwater pond. The length–weight relationships for the gurami sago reared in concrete ponds were shown by *W* = 7.9368 *L* ^1.0146^ (with *R* ^2^ = 0.83, Figure 3) and by *W* = 3.7760 *L* ^1.2641^ (with *R* ^2^ = 0.75, Figure 4) for the floating net cages and by *W* = 9.3106 *L* ^1.0056^ (with *R* ^2^ = 0.75, Figure 5) for the earthen freshwater ponds. The three *b-values* of each aquaculture system differed from 3.0 ( *b*<3, *p*<0.05) indicating negative allometric growth. The Fulton’s condition index in the concrete pond, floating net cages and earthen freshwater pond were 2.45, 1.91, and 3.36, respectively.

**Figure 6.**
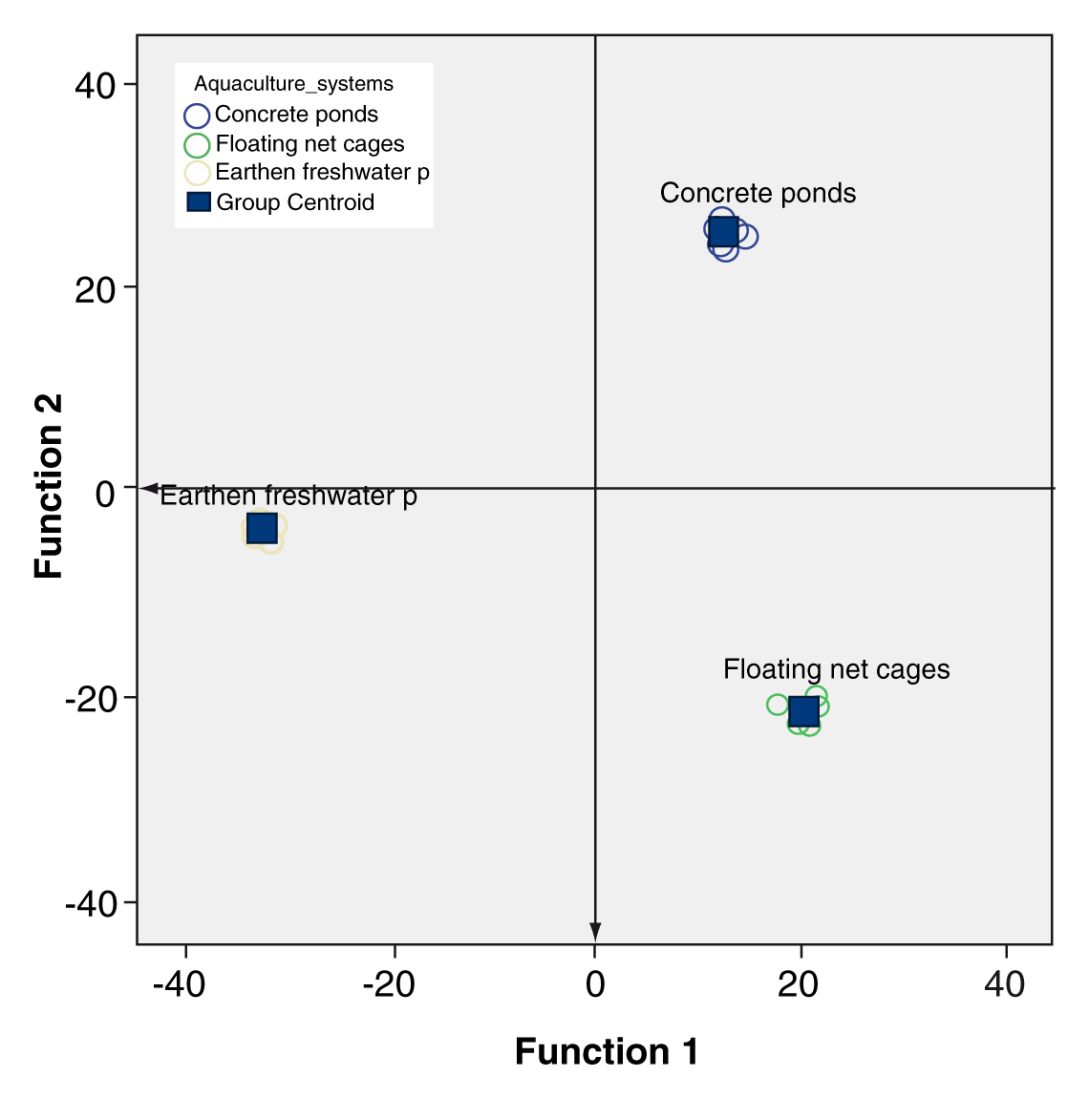
Sample centroids of discriminant function scores based on water quality parameters.

In this study, the water quality was recorded weekly from each aquaculture system during the experiment period and showed significant differences (p<0.05) in terms of TDS, TSS, DO, COD, BOD, ammonia, nitrites, nitrates, pH, alkalinity and hardness, only water temperature did not show a significant difference. Furthermore, in the principal component analysis, PC1 accounted for 66.67% of the 12 parameters of water quality, which had a positive correlation with all water quality parameters. This result shows that value has an effect on the water quality parameters in aquaculture systems. Alkalinity, hardness, pH, and dissolved oxygen make high contributions to the aquaculture system ( Table 3). The plot of PC1 and PC2 shows highly isolated water quality parameters between concrete ponds, floating net cages and earthen freshwater ponds ( Figure 6).

**Table 3.** Principal component loading and degree of divergence in quantitative traits among samples (Qst) of the water quality parameters.

Water quality parameters	PC1	PC2	Qst
Total dissolved solids	.959	.213	.965
Total suspended solids	.852	-.488	.964
Dissolved oxygen	-.896	-.409	.971
Biological oxygen demand _5_	.954	.228	.962
Chemical oxygen demand	.972	-.095	.955
Ammonia	.933	.252	.934
Nitrite	.840	-.208	.749
Nitrate	.222	.902	.862
Water temperature	.356	-.477	.354
pH	-.580	.788	.956
Alkalinity	.057	.989	.982
Hardness	.043	.982	.966

Extraction Method: Principal component analysis (PCA).

## Discussion

The aquaculture industry needs environmentally friendly aquatic ecosystems. Therefore, aquaculture practices must use aquaculture systems that minimize waste loads and increase added value ^1, 3, 38, 39^. In fact, the diversification of aquaculture systems with the efficient use of land resources can increase aquaculture production ^28, 40^. The comparisons between concrete ponds, floating net cages and earthen freshwater ponds are relevant to determine their relative per unit volume performance of juveniles-rearing of gurami sago. The rearing of gurami sago is an alternative diversity of aquaculture that can contribute to the development of commercial production in the future.

Gurami sago was successfully reared in concrete ponds, floating net cages and earthen freshwater ponds. However, their growth performance was best in the floating net cages. The high survival rate of gurami sago was found in the floating net cages, which was similar to the gurami tambago strain ^8^ and gurami sago in the artificial ponds lined with membranes ^12^. On the other hand, the survival rates of gurami sago in earthen freshwater ponds (89.44%) were higher than those of carps (65.74%) and stinging catfish (69.00%) in freshwater ponds ^27^.

The growth rate of gurami sago, with an average initial weight of 54.18 g, was faster in floating net cages than in concrete ponds and earthen freshwater ponds, with specific growth rate (SGR, % day ^-1^) values of 0.67, 0.75 and 0.62, respectively. In contrast, Budi *et al*. ^41^ stated that giant gourami belonging to the local gurami soang strain in the laboratory with initial weight of 15.83 g had faster growth with an SGR value of 2.13% day ^ˉ1^. The specific growth rate of fish seems to be influenced by the initial weight, strains and aquaculture systems. The economic AFCR value of fish fed in floating net cages was lower than that of fish fed in concrete ponds and earthen ponds. Therefore, it can reduce the cost of feed and increase the economic benefits to producers. This condition indicates that the culture of gurami sago in floating net cages gives fish a chance to consume more feed. However, this AFCR was lower than that of *Nile tilapia* ^42, 43^, and giant gourami ^8^, and higher than the African catfish AFCR value ^44^.

In this study, the growth performance of different gurami sago individuals in each aquaculture system was caused by differences in water quality. The PCA shows that there are differences in water quality among concrete ponds, floating net cages and earthen freshwater ponds. The alkalinity, hardness, and pH might affect the growth performance of gurami sago in aquaculture systems. Pouil *et al.* ^7^ state that nutrient input in the cultured of giant gourami in the earthen freshwater ponds strong correlation with sediment nutrient accumulation, of which 61% total nitrogen and 77% phosphorus inputs were trapped in the accumulated sediments, which directly impacts to aquatic environment. Furthermore, Boyd *et al*. ^45^ stated that the productivity of aquatic ecosystems and aquaculture production can be influenced by water quality, such as alkalinity, hardness and pH. Many studies have found that growth performance can be affected by water temperature ^46, 47^, DO level ^48^ and nitrite-nitrogen ^27^.

The aquaculture system influences the production of gurami sago. The highest production was found in the floating net cages, with a value of 3.36 kg m ^ˉ3^. However, its production was lower than that of other freshwater cages, for example 4.19 to 10.70 kg m ^ˉ3^ for the strain gurami tambago ( *O. goramy*) ^8^, 25.4 to 26.3 kg m ^ˉ3^ for pirarucu ( *Arapaima gigas*) ^49^, 88.5 kg m ^ˉ3^ for silver perch *,* ( *Bidyanus bidyanus*) ^50^ and 11.60 to 16.03 kg m ^ˉ3^ for spotted rose snapper ( *Lutjanus guttatus*) ^36^. It seems that different levels of aquaculture production can be influenced by species diversity, stocking density and duration of aquaculture. Giant gourami can produce a maximum profit after 324 days of aquaculture ^51^.

Herein, we recommend gurami sago strain aquaculture in concrete ponds, floating net cages and earthen freshwater ponds for 324 days. According to De Oliveira Continho *et al*, ^52^ fish reared in cages can increase the variation in weight production. In contrast, the freshwater cages have been marred by increasing the frequencies of fish mortality, causing negative implications to finances and the environment ^23, 24, 53^. Bosma and Verdegem ^54^ reported that the direct risks related to aquaculture in ponds were habitat destruction, suboptimal freshwater consumption, organic pollution, eutrophication, and water contamination with pesticides. These factors can cause production to decline and cause low economic value.

In this study, after the analysis of growth performance and production, we also analyzed the length–weight relationship and condition factor (K) from aquaculture systems. The exponent of the length–weight relationship - or per Froese ^55^, the allometric coefficient (b) - calculated was 1.0146 for concrete ponds, 1.2641 for floating net cages and 1.0056 for earthen freshwater ponds. Gurami sago grown in different aquaculture systems showed negative allometric growth. These values were smaller than 2.94 for the culture of *Tilapia zillii* ^56^ and 2.99 and 2.93 for *Pangasianodon hypophthalmus* and *Clarias gariepinus,* respectively ^57^. The K-values were not different among concrete ponds, floating net cages and earthen freshwater ponds. The finding explains that no different morphological factors were found in gurami sago cultures in concrete ponds, floating net cages and earthen freshwater ponds. However, cultures of gurami sago in floating net cages had a smaller condition factor or had values close to 1.00. The variation in the condition factor (K) of gurami sago may be influenced by different factors, such as environmental conditions, feed intake and increased of body weight. The condition factor (K) of fish depends on many factors, including species diversity, growth, physiological performance, age, and gonadal maturity ^14, 56, 58– 60^.

## Conclusion

In conclusion, our study showed that gurami sago strain can be efficiently reared in concrete ponds, earthen freshwater ponds and floating net cages. For all tested parameters, the best aquaculture system was found in the floating net cages. Nevertheless, further investigations on fish farming in the floating net cages which a technically feasible and economics at a larger scale are needed to determine commercial interest and environment impacts, especially on water quality, in an effort to develop of gurami sago fish farming in Indonesia.

## Data availability

### Underlying data

Figshare: Row data growth performance of gurami sago in different aquaculture systems.doc, https://doi.org/10.6084/m9.figshare.11719542.v1 ^61^.

This project contains the following underlying data:

– Table 1. Sample size of weight and length of the gurami sago strain (0 days, 30 days, 60 days and 90 days) in the concrete pond culture ( *N*=24)
– Table 2. Sample size of weight and length of the gurami sago strain (0 days, 30 days, 60 days and 90 days) in the floating net cage culture ( *N*=24)
– Table 3. Sample size of weight and length of the gurami sago strain (0 days, 30 days, 60 days and 90 days) in the earthen freshwater pond culture ( *N*=24)
– Table 4. Sample size means of initial weight, final body weight and weight gain of gurami sago ( *N*=24)
– Table 5. Sample size means of initial length, final total length and length increase of gurami sago ( *N*=24)
– Table 6. Data on mean initial biomass, final biomass and gross yield of gurami sago ( *N*=24)
– Table 7. Data on mean SGR, feed intake and apparent feed conversion rate of gurami sago ( *N*=24)
– Table 8. Data on mean economic food conversion, feed conversion efficiency and waste load of feed ( *N*=24)
– Table 9. Data on mean growth (g) of gurami sago at 0 days, 30 days, 60 days, and 90 days ( *N*=24)
– Table 10. Data on mean size distribution (g) of gurami sago in the different aquaculture systems in the 90-day trial ( *N*=72).
– Table 11. Row data for water quality parameters of reared gurami sago in different aquaculture systems for each month.

Figshare: Row Data_survival (fish) of gurami sago_12 Feb 2020.doc, https://doi.org/10.6084/m9.figshare.11845560.v1 ^62^

Data are available under the terms of the Creative Commons Attribution 4.0 International license (CC-BY 4.0).
