# Supplementary material for: Growth, production and feed conversion performance of the gurami sago (Osphronemus goramy Lacepède, 1801) strain in different aquaculture systems
Source: F1000Res. 2021 Jan 27;9:161. Originally published 2020 Mar 3. [Version 3] doi: 10.12688/f1000research.22201.3 (PMC7791349; doi:10.12688/f1000research.22201.3)
Supplement: Peer review history

# Peer review: Reviewer response for version 3

**DOI:** 10.5256/f1000research.53869.r78325

**Published:** 2021-02-03

**Authors:** Simon Pouil

**Affiliations:** 1 INRAE, Jouy-en-Josas, France

**General comment:**

 I carefully read this revised version of the manuscript and, unless I am mistaken, none of my previous comments have still be considered. No rebuttal and/or answer has been provided as well. So, for these reasons, I still have some reservations about the publication of this, moreover very interesting, study.

 I hope my previous comments would help the Authors to revise their manuscript.

Is the work clearly and accurately presented and does it cite the current literature?

Partly

If applicable, is the statistical analysis and its interpretation appropriate?

Partly

Are all the source data underlying the results available to ensure full reproducibility?

Yes

Is the study design appropriate and is the work technically sound?

Yes

Are the conclusions drawn adequately supported by the results?

Partly

Are sufficient details of methods and analysis provided to allow replication by others?

Partly

Reviewer Expertise:

aquaculture, aquatic ecotoxicology

I confirm that I have read this submission and believe that I have an appropriate level of expertise to confirm that it is of an acceptable scientific standard, however I have significant reservations, as outlined above.

# Peer review: Reviewer response for version 2

**DOI:** 10.5256/f1000research.25835.r74355

**Published:** 2021-01-07

**Authors:** Rudy Agung Nugroho

**Affiliations:** 1 Animal Physiology, Development, and Molecular Laboratory, Department of Biology, Faculty of Mathematics and Natural Sciences, Mulawarman University, Samarinda, Indonesia

I have read and compared between version 1 and version 2, the article has some improvement. There are some points that should be taking into attention for the authors for the next research and I can conclude that this article can be indexed after an amendment.

**ABSTRACT**

- **Methods:** A mean of 240 juveniles were stocked (mean, 54.53 g and 13.88 cm), please add the word (initial weight mean, 54.53 g and 13.88 cm).
- **Conclusion:** Please mentioned which production is better among different aquaculture systems.

**INTRODUCTION**

- Sufficient.

**MATERIALS AND METHODS**

- Sufficient.

**RESULTS AND DISCUSSION**

- Well written and organized.

**CONCLUSION**

- As mentioned in the summary: “our study showed that gurami sago strain can be efficiently reared in floating net cage”, in which term gurami can be efficient rear in floating net cage for all parameters or some? Please add information more briefly.

Is the work clearly and accurately presented and does it cite the current literature?

Yes

If applicable, is the statistical analysis and its interpretation appropriate?

Yes

Are all the source data underlying the results available to ensure full reproducibility?

Yes

Is the study design appropriate and is the work technically sound?

Yes

Are the conclusions drawn adequately supported by the results?

Yes

Are sufficient details of methods and analysis provided to allow replication by others?

Yes

Reviewer Expertise:

Fish nutrition, Animal Physiology, Biology Molecular

I confirm that I have read this submission and believe that I have an appropriate level of expertise to confirm that it is of an acceptable scientific standard.

# Response

**Published:** 2021-01-09

**Authors:** Hafrijal Syandri

**Affiliations:** Bung Hatta University, Indonesia

We have been added some information to complete our article :

**Response for Rudy Agung Nugroho Comments:**

**ABSTRACT :**

**methods:** We have been added the word initial weight means, 54.53 g and initial length 13.88 cm in the sentence

**Conclusion** : The best aquaculture system was found in the floating net cages.

**CONCLUSION :**

 In conclusion, our study showed that gurami sago strain can be efficiently reared in concrete ponds, earthen freshwater ponds and floating net cages. For all tested parameters, the best aquaculture system was found in the floating net cages.

# Peer review: Reviewer response for version 2

**DOI:** 10.5256/f1000research.25835.r62660

**Published:** 2020-09-11

**Authors:** Peter Vilhelm Skov

**Affiliations:** 1 DTU Aqua, Section for Aquaculture, The North Sea Research Centre, Technical University of Denmark, Hirtshals, Denmark

The desired corrections have been addressed. There are still some grammatical issues in the text, but I presume the editorial staff will handle this.

Is the work clearly and accurately presented and does it cite the current literature?

Yes

If applicable, is the statistical analysis and its interpretation appropriate?

Partly

Are all the source data underlying the results available to ensure full reproducibility?

Partly

Is the study design appropriate and is the work technically sound?

Yes

Are the conclusions drawn adequately supported by the results?

Partly

Are sufficient details of methods and analysis provided to allow replication by others?

Partly

Reviewer Expertise:

Aquaculture nutrition and bioenergetics

I confirm that I have read this submission and believe that I have an appropriate level of expertise to confirm that it is of an acceptable scientific standard.

# Peer review: Reviewer response for version 2

**DOI:** 10.5256/f1000research.25835.r62661

**Published:** 2020-05-12

**Authors:** Simon Pouil

**Affiliations:** 1 INRAE, Jouy-en-Josas, France

I acknowledge the Authors did revise their manuscript. Nevertheless, most of my previous comments remained not addressed in the revised version. The Authors should argue why they did not consider these comments. Please find below the comments, listed by sections, that should be addressed before submission of a new revised version.

**General comment:**

 I believe it is really important at this point that the Authors deeply revise Introduction and Materials and Methods taking into account all the comments of the reviewers to ensure that the objectives of the study are clear, that the experience is scientifically valid and that the data analysis has been performed correctly. This is why I am waiting for a thoroughly revised version before providing additional comments on the Results and Discussion.

**Introduction:**

 The flow of the Introduction may be improved. Thus, the first paragraph is not informative enough and too general. I suggest providing relevant figures about freshwater finfish production in Indonesia showing that this country is among the main producing country in the world. Some sentences could be added regarding the important role of freshwater aquaculture in the country by providing food and economic resources as well as employment in rural communities…

 Then the second paragraph should be focused on giant gourami as Authors did but maybe in a awkward way because the sentences said that giant gourami is important but this species accounts for only 7% of the national production. Even if it is true, giant gourami production represents now more than 100,000 tonnes/year of production and this production is growing (latest available production volumes and increasing rate per year should be provided). This species has high patrimonial and economic values (among the most expensive freshwater fish species in Indonesia). Instead of having the comparison of production volumes with other freshwater fish species, I suggest to the Authors to provide data and relevant arguments to highlight the importance of giant gourami in Indonesia. Most of the information should be available in the following references:

 FAO (2019) Cultured Aquatic Species Information Programme. Osphronemus goramy. Cultured AquaticSpecies Information Programme. Text by Caruso, D., Arifin, Z.O., Subagja, J., Jacques Slembrouck, J.and New, M. In: FAO Fisheries and Aquaculture Department [online]. Rome. Updated 26 September 2019

 FAO (2020). FishStatJ: software for fishery statistical time series. FAO, Roma.

 Thus, the structure of the Introduction could be, with appropriate transition sentences:

 1/ Importance of freshwater finfish aquaculture in Indonesia

 2/ Giant gourami among the main freshwater commodities in the country

 3/ Gaps of knowledge in gourami aquaculture

 4/ Rearing structures for giant gourami aquaculture

 5/ Objectives of the study

 In my previous report I highlighted the confusion between “strain” and “species” with those terms used interchangeably throughout the manuscript. Again, gurami sago is not a new species as the Authors said, it is a strain of giant gourami *Osphronemy goramy.* Here again, as I said on my previous report, I do not see the point to be focused on this strain in the Introduction because the findings from this study may be interesting for giant gourami in general. The strain used should be only mentioned in the Materials and Methods section.

**Materials and Methods:**

 In the subsection “Ethical considerations”, I think the sentence “The animals used in this study did not suffer during the experiment” should be deleted because it is always difficult to assess suffering in fish. The most important information regarding ethical considerations was already provided.

 As said in the previous review performed by Reviewer 2, water parameter values should be included in a Table within the manuscript, not as additional data.

 Change “Sampling design” by “Experimental procedures”

 In this subsection, when the origin of the fish is provided I suggest saying that the fish belonged to the “Gurame sago” strain, moving the information regarding this strain from the Introduction.

 I suggest rephrasing the following sentence “Twenty-four fish samples were obtained from each concrete pond, floating net cage and earthen freshwater pond. 10% of the fish were sampled every month for each aquaculture system, due to giant gourami is sensitive to handling.” to “Twenty-four fish were sampled monthly from each concrete pond, floating net cage and earthen freshwater pond. The sample size and sampling frequency were selected in order to sample 10% of the total number of fish for each rearing structure while limited stress for the fish caused by handling.”

 I am not sure to understand how the “Waste load of feed” and “FCE” were evaluated since they require to be able to quantify the feed ingestion, right? I do not know how such an estimation can be performed in the tested rearing systems. The Authors should clarify this point already highlighted in the previous report.

 For the data analysis, assumptions of ANOVA (normality and variance homoscedasticity) should be tested and met on residuals, it should be clearly indicated in the manuscript.

 “Levine’s test” should be corrected by “Levene’s test”.

 “One-way ANOVA was used to analyze the effect of each treatment”, effects on what?

 The Authors should be precise regarding the data analyzed through ANOVA. I guess all of them did not meet the assumptions for using parametric ANOVA.

 In the same section, the Authors said they used discriminant analysis to examine grouping of the rearing systems using water quality parameters but the terms “Principal Component Analysis (PCA)” were used in their response to the reviewer comments that are not referring to the same statistical approach. The actual analysis performed should be clarified and better described in the data analysis section by explaining potential data transformation prior to the analysis, how the principal components have been selected…

**Figures and Tables**

 As I said in my previous report, the Authors should provide visible standard deviations values and statistical differences in the Figures when it is appropriate and better axis scale in order to improve data readability. For allometric relationships, p-values for model estimates should be provided. Overall, the Authors should pay special attention to provide revised Figures allowing them to clearly visualize each data.

 Again, water quality parameter values should be provided in a Table within the manuscript.

Is the work clearly and accurately presented and does it cite the current literature?

Partly

If applicable, is the statistical analysis and its interpretation appropriate?

Partly

Are all the source data underlying the results available to ensure full reproducibility?

Yes

Is the study design appropriate and is the work technically sound?

Yes

Are the conclusions drawn adequately supported by the results?

Partly

Are sufficient details of methods and analysis provided to allow replication by others?

Partly

Reviewer Expertise:

aquaculture, aquatic ecotoxicology

I confirm that I have read this submission and believe that I have an appropriate level of expertise to confirm that it is of an acceptable scientific standard, however I have significant reservations, as outlined above.

# Peer review: Reviewer response for version 1

**DOI:** 10.5256/f1000research.24486.r60878

**Published:** 2020-04-01

**Authors:** Peter Vilhelm Skov

**Affiliations:** 1 DTU Aqua, Section for Aquaculture, The North Sea Research Centre, Technical University of Denmark, Hirtshals, Denmark

**Introduction:**

- “Aquaculture activities are responsible for the supply of fish for human consumption. To meet the demand for food from aquaculture production, competition uses natural resources, such as land and water ^1– 3^. Many studies state that aquaculture production depends on many factors, including its species, aquaculture system, technical efficiency, production inputs, and infrastructure” - This is a rather convoluted paragraph. Please rephrase to improve coherence.
- “In 2018, the total aquaculture production in Indonesia was 16,032,122 metric tons” - That is probably correct, but then you go on to state that it all comes from fish culture. I think (supported by FAO 2018 yearbook) that around 12 million tons is seaweed. Please check and correct.
- ”synthetic sheet ponds..” - I would prefer “artificial ponds lined  with membranes” or something similar
- It is not clear to me what is meant by ”middle-scale commercial culture”. Is this in relation to intensity? Please clarify.

**M&M:**

- “Feed conversion efficiency (FCE) = [fish weight gain (g)/total feed ingested (g)]
  Apparent feed conversion rate (AFCR) = supplied feed/increase fish weight” - It is interesting that you have two indicators of feed performance where one is listed as apparent. I presume that feed waste was not collected in any of your rearing systems, and therefore all of your feed intake are apparent and based on  “supplied feed”, also the FCE.
- “Waste load of feed = [feed intake (kg)] – [final biomass (kg)]” - I am not familiar with this variable, nor do I completely understand what it signifies, but presumably, it should be biomass gain, and not just final biomass?

**Results:**

- One of the things that can explain the observed differences in growth performance is likely to be your water quality parameters. While it is fine with the PCA plot, I would really like to see the water quality measurements in a table. Once these are available, perhaps it would be possible to discuss which water quality parameters would be essential to control to successfully produce gourami in land-based systems.

Is the work clearly and accurately presented and does it cite the current literature?

Yes

If applicable, is the statistical analysis and its interpretation appropriate?

Partly

Are all the source data underlying the results available to ensure full reproducibility?

Partly

Is the study design appropriate and is the work technically sound?

Yes

Are the conclusions drawn adequately supported by the results?

Partly

Are sufficient details of methods and analysis provided to allow replication by others?

Partly

Reviewer Expertise:

Aquaculture nutrition and bioenergetics

I confirm that I have read this submission and believe that I have an appropriate level of expertise to confirm that it is of an acceptable scientific standard, however I have significant reservations, as outlined above.

# Response

**Published:** 2020-04-12

**Authors:** Hafrijal Syandri

**Affiliations:** Bung Hatta University, Indonesia

**Introduction:**

- Introduction in first paragraph have been rephrase to improve coherences in the paragraph.
- We have omitted the data of Indonesian fisheries production in the introduction.
- “Synthetic sheet ponds” have been change to “artificial ponds lined with membranes”.
- “middle-scale commercial culture” meant “middle-scale farms”

**M & M**

- We used the “Feed Conversion Efficiency (FCE)” Formula based on Chatvijitkul *et al*. 2017.

Reference: Chatvijitkul, S., Boyd C.E., Davis, D.A., McNevin, A.A. (2017). Pollution potential indicators for feed-based fish and shrimp culture. Aquaculture 477: 43-49.

- We have been revised the formula of “Waste load of feed”. Please see in sub-bab “Measurement Parameters”.

**Results:**

 In Table 1, the parameters of waste load /kg of feed were added

 The water quality parameters have been revised. We have data of water quality parameter in Table form, and it is has been state in raw data which have uploaded in Figshare (see Table 11 revised).

# Peer review: Reviewer response for version 1

**DOI:** 10.5256/f1000research.24486.r60880

**Published:** 2020-03-19

**Authors:** Simon Pouil

**Affiliations:** 1 INRAE, Jouy-en-Josas, France

The present manuscript entitled "Growth, production and feed conversion performance of the gurami sago ( *Osphronemus goramy* Lacepède, 1801) strain in different aquaculture systems" examines the zootechnical performances of a giant gourami Osphronemus goramy strain called “gurami sago” in three different rearing structures: concrete ponds, floating net cages and earthen freshwater ponds.

 Overall, the study presented is quite simple but nevertheless interesting especially because some important gaps of knowledge remain in giant gourami aquaculture. Thus, results presented, showing better growth performance and feed conversion efficiency when giant gourami is reared in floating net cages, are relevant to the field. Having said that, I think that the present version of the manuscript may be improved in some ways.

 My main concerns are related to some methodological aspects as well as the presentation of the data.

 I addressed below some general comments regarding on the different sections of the manuscript. I hope the following comments help the authors in revising the manuscript.

**Introduction:**

 The description of the rationale of the study could be improved. I suggest to go straight to the point with a first paragraph explaining why giant gourami is important in Indonesian aquaculture and avoiding too general information. Authors may provide some production figures and explain, based on relevant references, that giant gourami is an emblematic local species with high practical and market value, omnivorous with a strong vegetarian component as thus, a candidate species for improving sustainability in aquaculture.

 Authors should clearly state that, although giant gourami has been reared for decades in Indonesia, there are still important gaps of knowledge in its aquaculture.

 Another point that is true throughout the manuscript: be careful in the use of “strain” and “species”. These two terms seem to be used as synonyms in the manuscript although they refer to different concepts. I am not sure that the focus done on the strain used is so important in the Introduction. I think that the results provided here are useful for the species itself and not only this specific strain.

**Methods:**

 Overall, I found the Methods well-presented and informative enough. Nevertheless, I have one important concern regarding the statistics. Indeed, water parameters were recorded monthly, meaning that only 3 values per rearing structures are available to perform the canonical discriminant functions (CDF). Considering the variations of most of the measured parameters that can be occur in rearing structures such as shallow earthen ponds sometimes on the same day, I think such analysis is not appropriate.

 Furthermore, Authors should state why only 10% of the fish were sampled every month. I guess is because giant gourami is sensitive to handling but this information may be interesting to add.

**Results:**

 As I already mentioned, I have some doubts regarding the validity of the CDF using water quality data.

 I believe that the presentation of the results can be improved. Authors should provide visible standard deviations values and statistical differences in the Figures when it is appropriate and better axis scale in order to improve data readability. For allometric relationships, p-values for model estimates should be provided.

 Table 1: “Final food conversion rate (Fulton’s K)”, I guess it should be changed by “Condition factor (Fulton’s K)”

**Discussion:**

 Although water quality is likely a key parameter to explain some of the observed differences in zootechnical performances among the rearing structures, unfortunately, since water parameter values were recorded only once a month, I think that there is not enough information provided to use these results.

**Conclusion:**

 In the concluding paragraph, I expected clear recommendations for giant gourami aquaculture based on the findings from this study.

**References:**

 I believe that some references relating to the aquaculture of the giant gourami are missing. I suggest to consider the following references which can be useful in the Introduction and Discussion:

 FAO (2019) Cultured Aquatic Species Information Programme. Osphronemus goramy. Cultured Aquatic Species Information Programme. Text by Caruso, D., Arifin, Z.O., Subagja, J., Jacques Slembrouck, J. and New, M. In: FAO Fisheries and Aquaculture Department [online]. Rome. Updated 26 September 2019.

 Arifin O.Z., Slembrouck J., Subagja J., Pouil S.,  Yani A., Asependi A., Kristanto A.H., Legendre M. (2020). New insights into giant gourami (Osphronemus goramy) reproductive biology and egg production control. Aquaculture 519: 734743. ^1^

 Kristanto A.H., Slembrouck J., Subagja J.,Pouil S., Arifin O.Z., Prakoso V.A., Legendre M. (2020). Egg and fry production of giant gourami (Osphronemus goramy): Rearing practices and recommendations for future research. Journal of the World Aquaculture Society 51: 119-138. ^2^

Is the work clearly and accurately presented and does it cite the current literature?

Partly

If applicable, is the statistical analysis and its interpretation appropriate?

Partly

Are all the source data underlying the results available to ensure full reproducibility?

Yes

Is the study design appropriate and is the work technically sound?

Yes

Are the conclusions drawn adequately supported by the results?

Partly

Are sufficient details of methods and analysis provided to allow replication by others?

Partly

Reviewer Expertise:

aquaculture, aquatic ecotoxicology

I confirm that I have read this submission and believe that I have an appropriate level of expertise to confirm that it is of an acceptable scientific standard, however I have significant reservations, as outlined above.

## References

[1] : New insights into giant gourami (Osphronemus goramy) reproductive biology and egg production control. *Aquaculture*.2020;519: 10.1016/j.aquaculture.2019.734743 10.1016/j.aquaculture.2019.734743

[2] : Survey on egg and fry production of giant gourami (Osphronemus goramy ): Current rearing practices and recommendations for future research. *Journal of the World Aquaculture Society*.2020;51(1) : 10.1111/jwas.12647 119-138 10.1111/jwas.12647

# Response

**Published:** 2020-04-12

**Authors:** Hafrijal Syandri

**Affiliations:** Bung Hatta University, Indonesia

**Introduction**:

The introduction has been improving. The paragraph go straight to the point of why giant gourami important in Indonesian Aquaculture.

**Methods:**

Overall, the method has been revised based on your comment. We also add water quality data which come from our daily logbook. Actually water quality parameters were recorded weekly, but at the first we show in the Table 3 only per month for results Principal Component Analysis (PCA). Now we have changed it into weekly recorded (Table 3).

**Results:**

The results have been revised based on your comment. The allometric relationships (p-values) have been added in Figures 3, 4 and 5. Furthermore, Table 1 has been revised.

**Discussion:**

The water quality parameters have been revised based on your comment. We have changed it into weekly recorded. We have data from our daily logbook which already recorded during the research period. The complete raw data shows in Figshare (Table 11 revised).

**Conclusion**

We have been revised the conclusion.

**References:**

We have been added some references based on your suggestion.
